# Evaluation of cortical venous drainage in patients with Acute Ischemic Stroke

**DOI:** 10.3389/fnins.2025.1557408

**Published:** 2025-04-01

**Authors:** Runyang Li, Xi Li, Jie Li, Xinjun Dai, Junhong Guo, Shaoshuai Wang

**Affiliations:** ^1^Department of Neurology, The First Hospital of Shanxi Medical University, Taiyuan, China; ^2^Department of Nephrology, Heping Hospital Affiliated to Changzhi Medical College, Changzhi, China; ^3^Department of Oncology and Hematology, Liuyang Hospital of Traditional Chinese Medicine, Hunan University of Chinese Medicine, Changsha, China

**Keywords:** Acute Ischemic Stroke, ischemic cerebrovascular disease, venous drainage, venous outflow, evaluation index, prognosis

## Abstract

The emergence of Mechanical thrombectomy (MT) has changed the treatment modalities for Acute Ischemic Stroke (AIS). But still 45 to 55% of patients cannot achieve functional independence after sufficient recanalization through endovascular treatment, defined as “futile reperfusion.” Poor collateral circulation and microcirculation are key factors affecting prognosis. In the past, the assessment of these mainly focused on intracranial arteries and their collateral, neglecting the important role of the venous system in acute brain injury. More and more studies have found that “poor venous drainage” is associated with poor prognosis. However, there is currently no unified standard for the assessment of “cortical venous drainage.” This paper reviews the pathophysiology of the relationship between “cortical venous drainage” and prognosis, as well as the assessment methods and indicators of “cortical venous drainage,” aiming to provide a strong basis for the preoperative assessment of AIS patients and the selection of treatment plans.

## Introduction

In clinical practice, it has been found that even up to half of the AIS patients still cannot achieve functional independence after MT (Consoli et al., 2023). “Collateral circulation and microcirculation” are considered to be the key factors affecting the prognosis of MT (Wang et al., 2023; Li et al., 2024). Good “collateral circulation and microcirculatory” can improve the prognosis of patients by reducing the core infarct volume and lowering the risk of postoperative hemorrhagic transformation (Li et al., 2024). “Venous drainage” (or venous outflow) is an evaluation of the downstream of microcirculation and the final outlet of cerebral blood circulation, and controls the local perfusion in the downstream area (Li et al., 2024), and good “venous drainage” reflects that blood flows unobstructed through brain tissue and is regarded as a powerful predictor of good micro-perfusion in brain tissue (Faizy et al., 2022). Multiple studies (Li et al., 2024; Faizy et al., 2022; Faizy et al., 2021a,b; Dai et al., 2024; Salim et al., 2025) have found that patients with “good venous drainage “have a better prognosis after MT treatment, therefore, for maintaining good perfusion of brain tissue, downstream venous outflow is as important as upstream arterial perfusion (Dai et al., 2024). The “cortical vein” covers the surface of the cerebral cortex like a network. It is a superficial part of the cerebral venous system and is mainly responsible for draining the blood of the cerebral cortex. Since the internal carotid artery system provides blood to the anterior 2/3 of the brain, the venous blood in this area is mainly drained through the cortical vein. Therefore, cortical venous drainage has a unique advantage in evaluating the collateral circulation in the anterior circulation blood supply area. Currently, the evaluation of cortical venous drainage is mostly based on imaging results, but the imaging techniques and indicators used in various evaluation methods are varied. This article conducts a detailed review of the pathophysiological mechanisms linking venous drainage to acute brain injury. Additionally, it elaborates on the assessment protocols for venous drainage that are established based on diverse imaging modalities. To further illustrate the discussed concepts, a selection of clinical cases is also presented in this article.

### Pathophysiology of cortical venous drainage and poor prognosis in acute ischemic stroke

Under normal circumstances, cerebral autoregulation matches the amount of blood entering the brain from the arterial system with that flowing out of the brain through the venous system. This prevents the brain from damage caused by hypoperfusion or hyperperfusion, keeping the total blood flow in the brain constant (Dai et al., 2024; Chen et al., 2015).

After acute stroke, the brain tissue in the infarcted area becomes edematous. The swollen end-feet of astrocytes can compress the thin walls of venules and capillaries, and even cause blood vessel occlusion. Due to the lack of a smooth muscle cell layer, the venous system is more prone to tissue edema and pressure elevation compared to the arterial system. The elevated cerebral venous pressure not only increases the exudate in the perivascular space but also leads to the disruption of the blood-brain barrier, increasing its permeability and further exacerbating cerebral edema (Chen et al., 2015). Clinically, in patients with a large core infarct volume, the occurrence of severe cerebral edema after thrombectomy may cause midline shift, affecting the prognosis (Nie et al., 2023).

Restoring blood supply to temporarily ischemic brain tissue may cause damage to the tissue bed, namely “ischemic reperfusion injury” (Nie et al., 2023). After acute brain tissue injury occurs, cerebral edema leads to an increase in intracranial pressure. This pressure compresses veins, obstructs venous outflow, causes local blood stasis, affects the blood supply to brain tissue and the excretion of metabolic products, resulting in the accumulation of metabolic waste and the generation of harmful free radicals. The subsequent inflammatory response and oxidative stress will exacerbate reperfusion injury (Salim et al., 2025; Nie et al., 2023). Abnormal “venous outflow” such as that of the superficial middle cerebral vein (SMCV) affects the blood supply and metabolism of tissues, aggravating tissue damage. This indirectly demonstrates the association between venous drainage disorders and reperfusion injury (Dai et al., 2024). On the other hand, when there is a mismatch between “decreased venous outflow” and “increased arterial blood flow” after blood vessel recanalization, venous pressure will rise. Venous hypertension may cause the rupture of fragile thin-walled cortical veins, leading to secondary cerebral hemorrhage, that is, “hemorrhagic transformation”.

Currently, it is believed that for clinical outcomes, tissue reperfusion is more important than arterial recanalization, although angiography shows complete recanalization, persistent tissue hypoperfusion due to incomplete microcirculation reperfusion (the “no-reflow”) can still lead to poor prognosis (Ng et al., 2022; Zaidat et al., 2013). The hypoperfusion mechanism in the “no-reflow” phenomenon is mainly due to pericyte contraction, endothelial cell swelling, lumen blockage by white blood cells and microthrombi, as well as damage to blood vessels caused by thrombectomy itself, resulting in impaired capillary bed perfusion (Ng et al., 2022; Pasarikovski et al., 2020).

When arterial collateral blood flow is interrupted due to factors such as edema, microthrombi, and vasospasm, resulting in poor perfusion of ischemic tissue, a decrease in venous outflow will further exacerbate brain tissue edema in this area. At the same time, tissue edema increases interstitial pressure, further raising the resistance of the venous drainage system. This affects the normal return of blood, causing poor excretion of metabolic products in brain tissue and ultimately aggravating the damage to brain tissue (Dai et al., 2024; Chen et al., 2015).

### Assessment of cortical venous drainage based on different imaging tools

#### Computed tomography angiography

The Cortical venous opacification evaluation score (COVES) based on computed tomography angiography (CTA) is the most commonly used evaluation scheme at present. COVES selects three representative venous outflow networks, namely the Superficial middle cerebral vein (SMCA), Vein of Labbe (VOL), and Sphenoparietal sinus (SPS), which can basically cover the venous drainage of the entire MCA region. This means that COVES can comprehensively delineate the venous blood return in the MCA region and has lower anatomical variability compared with other cortical veins, making it more convenient and accurate when conducting comparisons among different individuals (Jansen et al., 2018; Kiliç and Akakin, 2008). In addition, since CTA can clearly display the vascular structure, it provides a good image basis for COVES, especially single-phase CTA, and its wide availability in clinical practice enables COVES to conduct evaluations more conveniently based on these high-quality CTA images.

**Figure 1:**
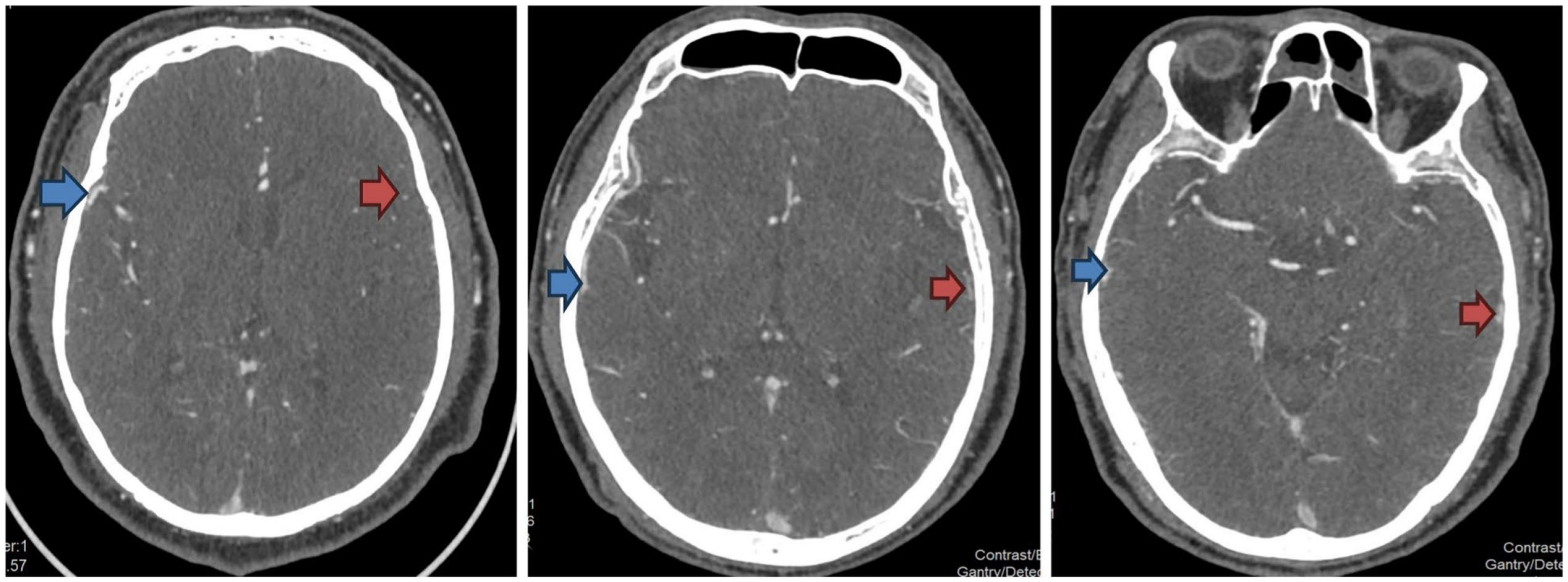
Examples of the CTA vein opacification score. The side indicated by the red arrow is the affected side, and the side indicated by the blue arrow is the healthy side. From left to right in the figure, the venous opacities are scored from 0 to 2 points, respectively.

The filling of each vein was graded by baseline CTA (Jansen et al., 2018) (Table 1, Figure 1). Patients with COVES>0 had a higher probability of good prognosis (mRS 0–2) (OR = 3.0; 95%CI:1.7, 5.4) (Faizy et al., 2022), however, there is still controversy regarding the optimal cut-off value for the COVES. In a multicenter retrospective study involving 649 patients, Faizy Tobias calculated the optimal cutoff value of COVES by ROC curve as 2.5 (area under curve 0.75; sensitivity 60%; specificity 79%) (Faizy et al., 2021a,b). In their study, COVES score of 0–2 was considered to indicate poor venous outflow, and COVES score of 3–6 was considered to indicate good venous outflow. And they also adopted this conclusion in another multicenter study involving 647 patients (Jansen et al., 2018). However, a study conducted by Gong et al. (2024) included a total of 332 patients, all of whom were large vessel occlusion patients receiving endovascular therapy in the late window period. Through marginal effect analysis, this study found that the probabilities of having a favorable prognosis (mRS: 0–2) and an unfavorable prognosis (mRS: > 2) intersected at a score of four. Patients with a COVES score ≥ 4 had a significantly higher probability of achieving a favorable prognosis than those with a COVES score < 4, suggesting that a COVES score of 4 might be the cut-off point for distinguishing a better prognosis.

**Table 1:** Comparison table of venous filling degree scores based on CTA.

	COVES			CVOS			VOS			PRECISE		
	Complete	Partial	Non	Complete	Partial	Non	Complete	Partial	Non	Complete	Partial	Non
SMCV	2	1	0	2	1	0	2	1	0	2	1	0
VOL	2	1	0	2	1	0	2	1	0	2	1	0
VOT	-	-	-	-	-	-	2	1	0	2	1	0
SPS	2	1	0	2	1	0	-	-	-	-	-	-
BVR	-	-	-	-	-	-	-	-	-	2	1	0
ICV	-	-	-	2	1	0	-	-	-	-	-	-
Effective drainage	≥1			≥4			≥4			≤3		
Poor drainage	<1			≤3			≤3			≥4		

Degree of contrast agent filling: Complete filling: the filling is similar to that of the contralateral hemisphere when compared with the contralateral side. Partial filling: Moderately filled when compared with the contralateral side. Non-filling: the density on the maximum-intensity-projection image is similar to that of the brain parenchyma, or the blood vessels are only visible due to blood stasis. COVES: Cortical venous opacification evaluation score; CVOS: comprehensive venous opacification score; VOS: venous opacification score; PRECISE: predicting recanalization effectiveness in cortical intravenous sinus embolism; SMCV: Superficial Middle Cerebral Vein; VOL: Vein of Labbé; VOT: Vein of Trolard; SPS: sphenoparietal sinus; BVR: basilar vein of Rosenthal; IVC: internal cerebral veins.

Although the COVES has been proven to be an independent predictor for ischemic cerebral perfusion and long-term prognosis (90-day) modified Rankin Scale (mRS) score, it can only assess the cortical venous drainage. The veins draining the MCA can be classified according to their drainage area: the superficial cortical area [superficial middle cerebral vein (SMCV) + vein of Trolard (VOT) + vein of Labbé (VOL) + basal vein of Rosenthal (BVR)] and the deep cortical area [internal cerebral vein (ICV) + thalamostriate vein (TSV)]. The three veins assessed by COVES only represent a part of the venous outlets in the MCA region. The arterial supply to the basal ganglia and striatum comes from the lenticulostriate arteries (LSA) arising from the M1 segment of the MCA. The venous drainage in this area mainly enters the ICV. However, it remains unclear whether assessing the degree of ICV opacification can provide more information for cortical venous drainage (Kiliç and Akakin, 2008; Adusumilli et al., 2023). Based on this, Adusumilli G proposed a comprehensive venous opacification score (CVO) that includes the ICV (Adusumilli et al., 2023) (Table 1). This study enrolled 648 patients who all underwent 90-day follow-up and had their mRS recorded. The study found that the success rates of recanalization procedures were similar between patients with CVO+ (4–8 points) and those with CVO- (0–3 points), both ranging from 50 to 95%. Nevertheless, compared with patients with CVO-, those with CVO+ were more likely to achieve functional independence at the end of the 90-day follow-up, and the correlation degree was significantly higher than that of the COVES score (−0.74 vs. −0.67, *p* = 0.006). This also indicates that a considerable proportion of recanalization in patients with CVO- may be ineffective, suggesting that the CVO score provides a more comprehensive assessment of cortical venous drainage.

In addition, since the SMCV ultimately drains into SPS, there is also controversy as to whether the mutual influence between them will reduce the effect of the COVES. Studies have found that only approximately 19.5% of patients have the dominant pattern of vein of VOL+ SMCV, while nearly 40% of patients have the dominant patterns of vein of VOT+ VOL, VOT + SMCV, or VOT + VOL + SMCV (Tanriverdi et al., 2009). Based on this, Li et al. (2024) proposed a venous opacification (VO) score that includes three veins, namely VOT, VOL, and SMCV. A total of 242 patients were enrolled in this study. The cut-off value for distinguishing good venous drainage was obtained through the AUCcurve as 3.5 points (AUC = 0.69; sensitivity 65%; specificity 73%). Defining a VO score of ≥4 points as “good venous outflow,” the results showed that good VO was associated with functional independence (mRS score 0–2 points) (odds ratio = 3.74, *p* < 0.001). However, this study was a single-center retrospective study. The optimal cut-off value of the VO score still needs to be verified in larger prospective cohort studies. Whether it has more advantages compared with the COVES also requires further verification.

Apart from the differences in the selection of draining veins, individual differences in venous conditions and the variability of superficial veins are also important factors that affect the evaluation of cortical venous drainage. Parthasarathy Rajsrinivas proposed the PRECISE (Predicting Recanalization Effectiveness in Cortical Intravenous Sinus Embolism) score for evaluating the contrast filling conditions of the SMCV, VOL, VOT, and BVR, based on the differences in cortical venous scores. The PRECISE score = the combined score of (SMCV + VOT + VOL + BVR) in the normal hemisphere – the combined score of (SMCV + VOT + VOL + BVR) in the affected hemisphere (Parthasarathy et al., 2013; Hoffman et al., 2019). This study demonstrated that the PRECISE score was significantly correlated with the arterial collateral status and infarct volume, and the PRECISE score of the affected hemisphere (rather than the combined score) was an independent predictor of prognosis. However, another retrospective study that included 103 patients compared the scoring efficacy of PRECISE and COVES and observed that, the COVES score was associated with prognosis (OR: 4.78; 95% CI 1.73–13.22), while there was no statistical significance between the PRECISE score and prognosis (OR: 1.02; 95% CI 0.37–2.80, *p* = 0.966). Therefore, the reliability of the PRECISE score still needs to be further verified (Hoffman et al., 2019).

#### Computed tomography

Non-contrast CT scan is a simple, reliable and systematic method for evaluating the early ischemic changes in the middle cerebral artery territory of patients. The Alberta Stroke Program Early CT Score (ASPECTS) is commonly used to predict the effect of thrombolysis and long-term prognosis (Puetz et al., 2009). Due to its poor ability to directly distinguish blood vessels, it is rarely directly used to assess cortical veins. In patients with ischemic stroke, the obstruction of cerebral microvascular perfusion can lead to the formation of ischemic cerebral edema. Moreover, the increase in net water absorption is an important sign of the formation of ischemic edema. Therefore, the method of measuring the density of the low-attenuation infarcted area by non-contrast head CT scan can be used. This method can directly quantify the net water uptake (NWU) of ischemic tissues (Faizy et al., 2021a,b) which helps to evaluate the progression of the disease and the treatment effect. Faizy TD conducted a study based on this principle. By studying the relationship between venous outflow (VO) and tissue NWU after the treatment of acute ischemic stroke (AIS) caused by large vessel occlusion, they found that a favorable VO curve (COVES score of 0–2) was correlated with less NWU in the ischemic tissue, and both favorable VO (odds ratio [OR]: 4.1; *p* < 0.001) and reduced NWU in the ischemic lesion (OR: 0.77; *p* < 0.001) were associated with a favorable functional outcome (modified Rankin Scale [mRS]: 0–2). This study provides a basis for using non-contrast CT scan to assess the drainage status of cortical veins in patients who cannot have computed tomography angiography (CTA) completed in a timely manner.

#### Magnetic resonance venography

Magnetic Resonance Venograph (MRV) is a non-invasive magnetic resonance angiography method, which is used to study diseases of the cerebral venous system. Selecting three veins in the COVES criteria and evaluating the venous system and prognosis according to their imaging manifestations in MRV images is also a feasible option. Currently, MRV imaging techniques include Phase-Contrast (PC), Time-of-Flight (TOF), and Three-Dimensional Contrast-Enhanced MRV (CE-MRV) (Pui and Cerebral, 2004; Paoletti et al., 2016). Since the blood flow velocity in the intracranial veins is much lower than that in the arteries, when TOF venography is adopted, the vascular display effect is usually poor. Therefore, the PC method or CE-MRV method is often used. In the PC method, the blood vessels show high-intensity signals on the original images, and then the Maximum Intensity Projection (MIP) images and 3D MIP images are reconstructed through algorithms (Paoletti et al., 2016). However, since only a few cases of ischemic stroke are caused by sinus venous or cortical venous thrombosis, in clinical practice, the arterial system is usually evaluated first when a disease occurs (Boehme et al., 2017). Compared with CTA examination which can evaluate both arteries and veins simultaneously, due to the different principles of arterial and venous imaging and the different sequences used in MRI, simply performing MRV can only provide venous information, which limits its application in the cerebrovascular evaluation of patients with Acute Ischemic Stroke (AIS).

#### Dynamic contrast enhanced MRI

Dynamic contrast enhanced magnetic resonance (DCE-MRI) is a unique quantitative MRI technique. It reflects tissue perfusion and vascular permeability through the enhancement and distribution process of gadolinium contrast agents within tissues, and can provide more information on vascular functions, enabling a more comprehensive assessment of the nature and extent of lesions.

The data of vascular imaging (DCE-MRA) or perfusion imaging (DSC-MRP) are stored in a 4D matrix, and MIP (Maximum Intensity Projection) images are created for each time point. By plotting the signal changes in the region of interest (ROI), the “signal intensity-time curves” of the non-ischemic hemisphere, the superior sagittal sinus, and the arteries and veins in the target area on the middle cerebral artery (MCA) can be obtained. According to the passage state of gadolinium contrast agents, the 4D image sets of DCE-MRA and DSC-MRP can be divided into the following four stages:

1. Arterial phase: It starts from the time when the contrast agent reaches the middle cerebral artery (MCA) until the peak of arterial injection.
2. Capillary phase: It ranges from just after the peak of arterial injection to before the peak of venous injection in the superior sagittal sinus.
3. Early venous phase: The first half of the venous phase, starting from the peak of venous injection to the starting point of the venous plateau.
4. Late venous phase: The second half of the venous phase (Roh et al., 2019; Kim et al., 2020). The collateral perfusion grading system is defined using the Magnetic Resonance Acute Ischemic Stroke Collateral (MAC) score (Table 2). Kim et al. (2020) and others evaluated 154 patients with acute ischemic stroke (AIS) caused by anterior circulation vascular occlusion and stenosis using DCE-MRA. There was a significant linear negative correlation between the MAC collateral perfusion grade and the mRS score in these patients (*p* < 0.001). A MAC score of ≥3 was independently associated with a favorable functional outcome (mRS: 0–2) (MAC grade 3: Odds Ratio = 27, *p* = 0.001; MAC grade 4: Odds Ratio = 17, *p* = 0.007; MAC grade 5: Odds Ratio = 27, *p* = 0.007).

**Table 2:** MR-based collateral score for acute ischemic stroke.

Points	Vascular manifestations
5	Regardless of the appearance in the arterial phase images, as long as there is no/slight delay in the collateral perfusion imaging during the capillary phase in the territory supplied by the middle cerebral artery.
4	The delay in the collateral perfusion imaging during the capillary phase is less than or equal to one-half of the territory supplied by the middle cerebral artery, and there is no/slight delay in the collateral perfusion imaging during the early venous phase.
3	The delays in the collateral perfusion imaging during both the capillary phase and the early venous phase are less than or equal to one-half of the territory supplied by the middle cerebral artery.
2	The delay in the collateral perfusion imaging during the capillary phase is greater than one-half of the territory supplied by the middle cerebral artery, while the delay in the collateral perfusion imaging during the early venous phase is less than or equal to one-half of the territory supplied by the middle cerebral artery.
1	The delay in the collateral perfusion imaging during the early venous phase is greater than one-half of the territory supplied by the middle cerebral artery, and the delay in the collateral perfusion imaging during the late venous phase is less than or equal to one-half of the territory supplied by the middle cerebral artery.
0	The delay in the collateral perfusion imaging during the late venous phase is greater than one-half of the territory supplied by the middle cerebral artery (regardless of how it is in other phases).

Although the techniques of DCE-MRI for evaluating cerebral perfusion and collateral circulation have been relatively mature, and compared with computed tomography angiography (CTA), it has less radiation, its limitations in spatial and temporal resolution make it unable to fully capture the flow and change process of the contrast agent within veins. Moreover, some small deep veins cannot be accurately identified and evaluated. In addition, due to individual differences, uneven distribution of contrast agents caused by blood flow velocity and the subjectivity of image processing, DCE-MRI has not been widely used in the evaluation of clinical AIS patients at present, and its application in this field still needs to be further explored.

#### Ultrasound

Due to the obstruction of the skull, it is difficult for ultrasound to clearly display the structures and blood flow conditions of the intracranial veins, which makes ultrasound rarely directly applied to the evaluation of intracranial veins. However, it has advantages in evaluating the internal jugular vein (IJV). The internal jugular vein is anatomically constant and easy to locate. It drains more than two-thirds of the blood flow of the superficial and deep cerebral venous systems through the confluence of sinuses, transverse sinuses, and sigmoid sinuses, and it is the main drainage pathway for the supply area of the middle cerebral artery (Mumtaz and Singh, 2019; Rhoton, 2002). Therefore, the blood flow state of the internal jugular vein can reflect the blood flow situation of the entire intracranial venous system to a certain extent.

Shang et al. (2022) conducted a retrospective study to evaluate the outflow of the internal jugular vein in 78 patients with acute anterior circulation stroke who had successful recanalization [the evaluation criteria for the outflow of the IJV (including the brachiocephalic vein) are shown in Table 3]. The cortical venous drainage was evaluated by the COVES score of the original head CTA images at admission. This study found that patients with poor bilateral IJV outflows had a worse functional prognosis. Poor bilateral IJV outflow is an independent risk factor for poor clinical prognosis (OR 17.843; *p* = 0.006), and poor ipsilateral IJV outflow is an independent predictor for hemorrhagic transformation (HT) (OR 3.708; *p* = 0.024).

**Table 3:** Evaluation of internal jugular vein drainage.

Staging	Unilateral internal jugular vein (including brachiocephalic vein)	Bilateral internal jugular veins
Effective drainage	The IJV (Internal Jugular Vein) is normal or mildly stenotic, with a stenosis rate of less than 50%.	Bilateral IJV is normal or with a stenosis less than 50%
Moderate drainage	-	One side of the IJV is normal or with a stenosis less than 50%, while the other side of the IJV has a stenosis of 50% or more or is occluded.
Poor drainage	Moderate-to-severe stenosis or the absence of IJV (Internal Jugular Vein), stenosis of 50% or more, or occlusion.	Stenosis of 50% or more or bilateral IJV occlusion.

IJV: Internal Jugular Vein.

The results of this study also provide a new idea for the application of ultrasound in the screening before thrombectomy and prognosis evaluation of patients with AIS. Although the results of this study still need to be verified in larger prospective cohorts, the rapidity, convenience, economy, non-invasiveness, and repeatability of ultrasound examination make its application prospects worthy of expectation.

#### Digital subtraction angiography

Digital Subtraction Angiography (DSA) is the gold standard for evaluating cerebrovascular stenosis and is widely used in the assessment of cerebrovascular status. It also has its unique advantages in the evaluation of venous drainage. DSA can display the process of venous drainage in real time and dynamically. When there are lesions in the venous drainage system, it can accurately locate the position of the lesions. Its high resolution helps to precisely observe the diameter of veins and the branching. A study by Consoli Arturo proposed a protocol for evaluating the collateral circulation venous phase (circulation venous phase CVP) based on angiography and used it as a predictive factor and a potential evaluation tool for the prognosis of patients with large vessel occlusion (LVO) in mechanical thrombectomy (MT) treatment (Consoli et al., 2023). In this study, the Anterior Segmental Intraparenchymal Transient (ASITN)/Society of Interventional Radiology (SIR) score was used to grade the anterior–posterior (AP) and lateral projections of the arterial collateral circulation. An ASITN/SIR grade > 2 was defined as good collateral circulation. CVP was defined as the visualization of the subcortical veins in the collateral circulation area-the area in the blood-supplying region of the occluded artery where collateral circulation compensation occurs. These veins are visible in the anterior–posterior projection and are vertical centrifugal veins that drain the subcortical and cortical blood flow in the occluded arterial area and are tributaries of the cortical veins draining into the superior sagittal sinus or the transverse sinus (Consoli et al., 2023; Shang et al., 2022). By conducting a binary classification of CVP (present or absent) to evaluate the venous collateral circulation and using the ASITN/SIR score (good or bad) to evaluate the arterial collateral circulation, a comprehensive assessment of the arterial and venous collateral circulation was carried out. The study found that compared with the ASITN/SIR score alone or CVP alone, the comprehensive evaluation of ASITN/SIR + CVP had a stronger correlation with the clinical prognosis. According to the ASITN/SIR grading, among patients with good arterial collateral circulation, the presence of CVP (+) was closely related to a good clinical outcome (modified Rankin Scale [mRS]: 0–2: 77.3% vs. 7.9%, *p* < 0.0001; mortality: 9.3% vs. 26.3%, *p* = 0.02), while the ASITN/SIR score alone had no significant correlation with the clinical prognosis (Consoli et al., 2023) This suggests that the evaluation of the venous phase based on angiography can make a great contribution to the assessment of collateral circulation. The high temporal and spatial resolution and real-time dynamic imaging of DSA give it greater advantages compared to ultrasound, computed tomography angiography (CTA), and magnetic resonance imaging (MRI). However, its high cost, invasive nature, and the limitations of contrast agents on the user population have always restricted its further application.

## Case report

### Case 1

**Figure 2:**
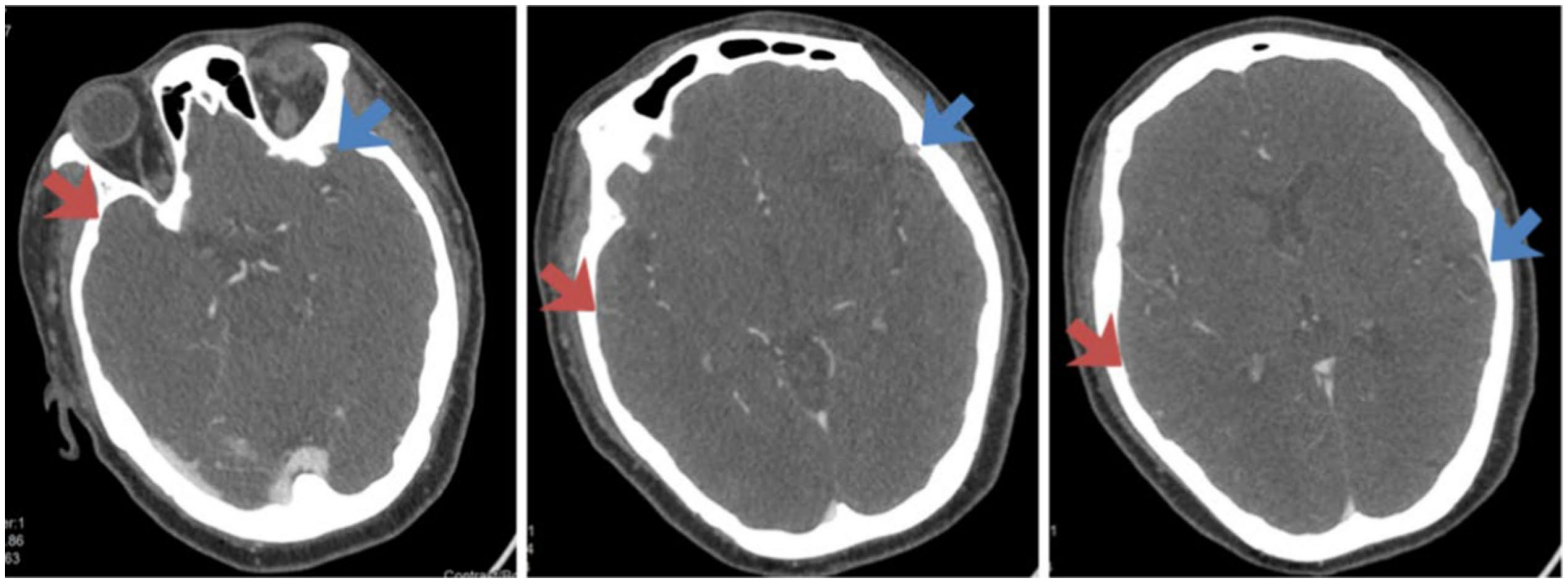
Assessment of venous outflow on the CTA images of Case 1. The side indicated by the red arrow is the affected side, and the side indicated by the blue arrow is the healthy side. From left to right in the figure, the COVES scores are as follows: SPS is 0 point, SMA is 1 point, LA is 0 point, and the total score is 1 point.

A 48-year-old female was found by her family members to have fallen to the ground at 7: 30 am. She presented with left-sided limb weakness, both eyes gazing to the right, and was unable to speak (Figure 2 shows the imaging pictures of this patient).

Pre-operative assessment: The NIHSS score was 20 points (2 points for level of consciousness +2 points for questions +2 points for commands +2 points for gaze +1 point for facial palsy +8 points for limbs +3 points for speech). Cerebral angiography was performed at 6:10 pm. During the operation, occlusion of the right internal carotid artery and the right middle cerebral artery was observed, with a pre-operative TICI grade of 0. Thrombectomy of the carotid artery and intracranial artery was carried out, and recanalization was achieved smoothly. The post-operative TICI grade was 2b.

After the operation, the patient developed bleeding in the right basal ganglia area and was transferred to the intensive care unit. After two weeks of treatment, her condition stabilized, and was transferred to the rehabilitation department for further treatment. At the time of discharge, she still had symptoms such as choking while drinking, inability to speak, and inability to lift her left-sided limbs. Thereafter, the patient regularly underwent rehabilitation training. At the 90-day follow-up, the patient was still unable to walk independently and required a great deal of assistance from others for daily activities such as eating, washing, and dressing. The mRS score was 4 points.

### Case 2

**Figure 3:**
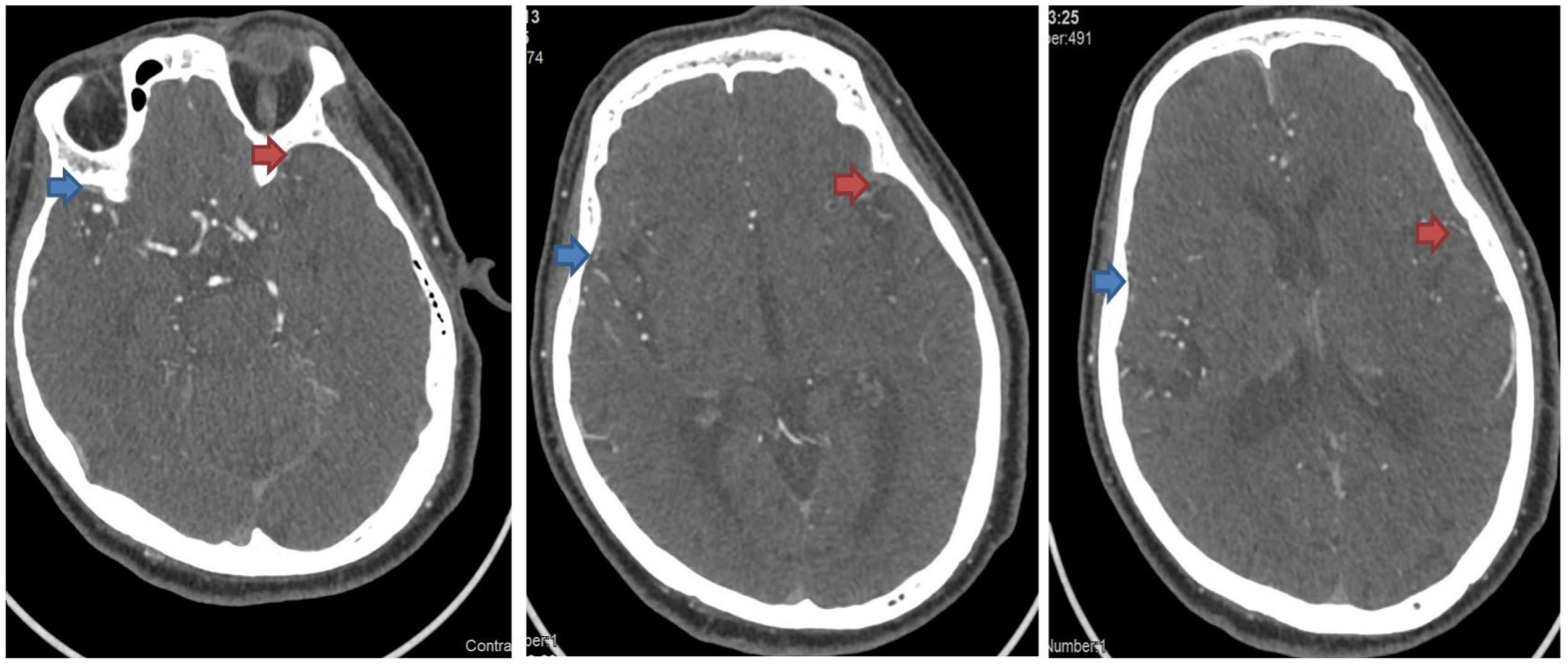
Assessment of venous outflow on the CTA images of Case 2. The side indicated by the red arrow is the affected side, and the side indicated by the blue arrow is the healthy side. From left to right in the figure, the COVES scores are as follows: SPS is 2 point, SMA is 1 point, LA is 2 point, and the total score is 5 point.

A 57-year-old male had no symptoms before going to bed. At 3:20 am, when he got up to use the toilet, he found that his right-sided limbs were numb and weak, he could not stand up, his speech was slurred, and he had urinary incontinence (Figure 3 shows the imaging pictures of this patient).

Pre-operative assessment: The NIHSS score was 10 points (1 point for gaze +7 points for limbs +1 point for sensation +1 point for speech). Cerebral angiography was performed at 7:30 am. During the operation, occlusion of the M1 segment of the left middle cerebral artery was observed, with a pre-operative TICI grade of 0. Intracranial artery thrombectomy was carried out, and recanalization was achieved smoothly. The post-operative TICI grade was 2b. The patient was discharged one week after the operation. At the time of discharge, his symptoms had basically recovered, and he could walk independently. At the 90-day follow-up, the patient occasionally had mild limb weakness, but he could take care of his daily life completely independently, with an mRS score of 1 point.

## Conclusion and prospect

Currently, the predominant approach for evaluating cortical venous drainage remains the Cortical Vein Opacification Score (COVES) based on Computed Tomography Angiography (CTA). It enjoys the broadest application and has yielded the most research outcomes. Although diverse improved scoring systems have been developed based on the COVES score, none of them has attained a comparable level of acceptance. 4D-CTA is being increasingly utilized in clinical practice owing to its enhanced temporal resolution. However, given the paucity of current research data, its evaluative value still awaits further investigation. Owing to the disadvantages at the technical principle level, both Computed Tomography (CT) plain scan and ultrasound have limited applications in the evaluation of cortical venous drainage. Nevertheless, considering their extensive prevalence and convenience which can supply a substantial amount of sample data, their values for further development and utilization remain highly significant. The Magnetic Resonance Imaging (MRI) examination is characterized by a relatively long duration, which significantly restricts its application in the preoperative assessment of patients with acute ischemic stroke, a situation where time is of the essence. Indeed, various versions of clinical guidelines uniformly refrain from recommending the use of MRI for such preoperative evaluations. Digital Subtraction Angiography (DSA), on the other hand, currently represents the gold standard for assessing cerebrovascular conditions and is an indispensable procedure prior to mechanical thrombectomy. Nevertheless, its value in the context of venous assessment remains to be fully explored, with numerous research directions still meriting in-depth investigation.

Reducing the incidence of “ineffective recanalization” after mechanical thrombectomy has been a focal issue in acute stroke interventional treatment in recent years. Comprehensive and effective pre-operative assessment may be one of the solutions. The difference in prognosis between the above two cases also confirms this issue to some extent. When a patient has obvious poor venous drainage, it is necessary to more carefully evaluate whether they meet the indications for mechanical thrombectomy.
